# Supplementary material for: IBD risk loci are enriched in multigenic regulatory modules encompassing putative causative genes
Source: Nat Commun. 2018 Jun 21;9:2427. doi: 10.1038/s41467-018-04365-8 (PMC6013502; doi:10.1038/s41467-018-04365-8)
Supplement: Supplementary file 2 — Description of Additional Supplementary Files [file 41467_2018_4365_MOESM2_ESM.pdf]

Description of Additional Supplementary Files

File Name: Supplementary Data 1
Description: List of 23,650 cis-eQTL (FDR < 0.05) detected in the CEDAR cohort.

File Name: Supplementary Data 2
Description: List of cis-acting regulatory modules (cRM) detected in the CEDAR cohort

File Name: Supplementary Data 3
Description: List of genes controlled by the same cRM in multiple tissues yet in opposite direction ($\vartheta < -0.6$).

File Name: Supplementary Data 4
Description: List of EAP with $|\vartheta| \geq 0.6$ with a DAP for either CD (sheet 1) or UC (sheet 2).

File Name: Supplementary Data 5
Description: Genes with reported eQTL effect potentially related to GWAS association signal for IBD that were not selected in this work.

File Name: Supplementary Data 6
Description: List of 4,476 variants detected by resequencing 521 coding exons of 42 candidate genes in 6,597 CD cases and 5,502 controls.

File Name: Supplementary Data 7
Description: Results of gene-based and module-based burden tests for rare (MAF < 0.005) disruptive (NS = LoF + damaging missense) and synonymous (S) variants.

File Name: Supplementary Data 8
Description: Primers used for targeted exon sequencing.
