# Supplementary material for: IBD risk loci are enriched in multigenic regulatory modules encompassing putative causative genes
Source: Nat Commun. 2018 Jun 21;9:2427. doi: 10.1038/s41467-018-04365-8 (PMC6013502; doi:10.1038/s41467-018-04365-8)
Supplement: Supplementary file 11 — Supplementary Information [file 41467_2018_4365_MOESM11_ESM.pdf]

Supplementary Information

IBD risk loci are enriched in multigenic regulatory modules encompassing causative genes.

Momozawa et al.

Supplementary note 1: Genes with strong DAP-EAP correlation

IL18R1 encodes the IL-18r1, the receptor of IL-18, a potent proinflammatory cytokine governing host-microorganism homeostasis and is postulated to play a role in IBD[1,2]. However, IL-18/IL-18r1 precise contribution to the disease remains controversial. Indeed, compared to wild-type mice, *$Il18^{-/-}$* and *$Il18r1^{-/-}$* full KO mice are more susceptible to AOM/DSS-induced colitis and polyp formation[3]. However, targeted deletion of *$Il18^{-/-}$* and *$Il18r1^{-/-}$* in intestinal epithelial cells confers protection from colitis and mucosal damage in mice[4]. In human, several studies have associated circulating or local IL-18 with IBD severity, suggesting that IL-18 could be an effector cytokine in IBD[5].

IL6ST encodes the interleukin 6 signal transducer protein (IL6ST), also called IL6 beta, GP130 or CD130. IL6ST is a common transmenbrane receptor for all family members of IL6 that include IL-6, IL-11, ciliary neurotrophic factor (CNTF), cardiotrophin-1 (CT-1), cardiotrophin like cytokine (CLC), leukaemia inhibitory factor (LIF), oncostatin M (OSM), neuropoitin (NPN) and interleukin-27 (IL-27)[6]. IL6 family members / IL6ST signaling pathways involve the activation of JAK (Janus kinase) family members, leading to the activation of STAT (signal transducers and activators of transcription) family, as well as the activation of MAPK (mitogen-activated protein kinase) pathway. These pathways are involved in cell survival, apoptosis, differentiation and proliferation[6]. The involvement of IL6/IL6ST/STAT3 in the pathophysiology of IBD is well documented[7]. Indeed, high circulating levels of IL6 is associated with increased severity of the disease[7]. T cells from IBD patient show increased STAT3 activation with increased expression of IL6ST and enhanced resistance to apoptosis[8]. A pilot clinical trial (phase I) targeting of IL6/IL6ST pathway in patients with CD has shown that blocking this pathway has effects similar to the inhibition of TNF[9,10].

THEMIS encodes the thymocyte-expressed molecule involved in selection (THEMIS), the expression of which is limited to lymphoid tissues. In mice, THEMIS is highly expressed in pre-TcR thymocytes and plays an important role in T-cell development and TCR activation signaling[11,12]. Its expression is reduced in differentiated T lymphocytes[12]. THEMIS deficiency in mice is associated with the

presence of higher percent of T_{reg} cells, with reduced TCR-mediated T cell response, increased proportion of memory CD4 and CD8 T cells and reduced proportions of naïve-phenotype populations[12]. Interestingly, all these T cells associated feathers are implicated in the pathogenesis of IBD. Indeed, lamina propria T cells in IBD are hypo-responsive to TCR stimulation and high number of effector T cells are present in the inflamed bowel[13]. As for T_{reg}, only moderate expansion was seen in intestinal lesions of Crohn's patients suggesting that their suppressive activity is probably not sufficient against the overwhelming effector T cells activity[13].

APEH encodes the acylpeptide hydrolase (APEH) enzyme that contributes to protein degradation processes in concert with the proteasome. It catalyzes the removal of *N*-acylated amino acids from acetylated peptides[14]. Its physiological role is not well undertood. SNPs in APEH gene hves been associated with both CD and UC[15]. Like other ubiquitin proteasome systems (UPS) such as USP40 or CYLD, APEH may also regulate the NF-kB pathway. Under this scenario, an alteration of NF-kB signaling may lead to aberrant immune response and inflammation.

ANKRD55 encodes an Ankyrin repeat domain-containing protein 55 with unknown function. Ankyrin repeats are composed of 33-34 aa and are the most abundant motifs in nature with highly diverse cellular functions[16]. SNPs at the ANKRD55 locus have also been associated with multiple sclerosis[17] and RA[18].

CISD1 gene encodes a highly conserved iron-sulfur domain-containing protein A, known as mitoNEET. This iron-containing protein is a dynamic redox-sensitive molecule that serves an important role in mitochondrial functions. It participates in critical process such as electron shuttling through the electron transport chain, regulation of enzymatic activity, and synthesis of heme and iron-sulfur clusters[19,20]. Deregulation of iron metabolism and associated anemia has been associated with IBD[21]. The role that mitoNEET plays in the etiology of IBD remains to be determined.

CPEB4 gene encodes the cytoplasmic polyadenylation element-binding protein 4 (CEBP4), which belongs to a family of proteins that bind mRNAs and contain a cytoplasmic polyadenylation element (CPE) in their 3′-UTR. Binding results in 3′-

poly(A) tail extension and translational upregulation of target mRNAs. *Cpeb4* mRNA is rhythmically regulated in mouse liver, conferring temporal translational regulation. In the absence of CPEB4, a large number of mRNAs are transcribed, but remain untranslated until needed[22]. A recent study, using knockout mice models, showed that CPEB4 was required for translation of numerous proteins involved in ER homeostasis and CPEB4 loss resulted in mitochondrial dysfunction and defective lipid metabolism, two hallmarks of ER stress. *Cpeb4* KO livers were highly susceptible to ER stress-induced apoptosis and to development of NAFLD[23]. In CD, reduced CPEB4 may also lead to ER stress and mitochondrial dysfunction.

DOCK7 encodes dedicator of cytokinesis 7 protein (Dock7), a member of Dock proteins family and an activator of Rac GTPases. DOCK7 plays an important role in axon outgrowth, Schwann cell migration, and axon myelination[24]. Mutation in this gene in mice leads to hypopigmentation suggesting a non-redundant role in the distribution and function of dermal and follicular melanocytes. However, mutant mice show normal neuronal function despite the high expression of DOCK7 in the developping brain, suggesting redundancy with other Docks[25]. The role of DOCK7 in IBD and immune cells function is totally unknown.

ERAP2 gene encodes an endoplasmic reticulum aminopeptidase (ERAP2), an enzyme involved in trimming of peptides for MHC-I loading. Aberrant ERAP2 function could influence peptide-HLA-B27 stability, formation of MHC-I free heavy chains and ER stress[26,27,28]. SNPs in *ERAP2* gene have been associated with CD[29]. Although the underlying mechanisms are not known, it is possible that ERAP2 modification contributes to the reported reduction of MHCI on CD4 T cells from CD patients[30]. ERAP2 modification may also contribute to the epithelial ER stress associated with CD and UC.

GNA12 encodes Guanine nucleotide-binding protein subunit alpha-12 or $G\alpha_{12}$, which belongs to the heterotrimeric G proteins. $G\alpha_{12}$ is found in tight junctions (TJ) where it interacts with ZO-1[31] and plays important roles in para-cellular permeability[32,33]. $G\alpha_{12}$ is ubiquitously expressed and interacts, upon receptor-mediated activation, with certain Rho guanine nucleotide exchange factors (RhoGEFs) which in turn mediate activation of the small GTPase RhoA[34]. Intestinal

permeability and barrier dysfunction is a hallmark of CD and UC. Several studies reported changes in the expression of several TJ proteins in both diseases[35]. It is conceivable that modifications in the $G\alpha_{12}$ pool leads to alteration of intestinal permeability. Tissue-specific $G\alpha_{12}$-deficient mice revealed important functions of this protein in modulating T cell trafficking and proliferation, as well as in the response to foreign and self antigens[36], important processes that may affect susceptibility for T cell-mediated diseases.

GPX1 encodes the glutathione peroxidase 1 (GPX1), a highly abundant and ubiquitously expressed cytosolic enzyme. Like all glutathione peroxidases family members, GPX1 catalyzes the reduction of H2O2 by glutathione and consequently, protects cells from oxidative damage. In IBD, it is believed that intestinal and colonic injuries and dysfunction is at least partially due to elevation of reactive metabolites of oxygen and nitrogen[37]. Although the role of GPX1 is not known in IBD, deficiency of both GPX1 and GPX2 in mice lead to spontaneous ileo-colitis and intestinal cancer[38]. A protective role of GPX1 and GPX2 against oxidative stress has also been suggested by studies reporting elevated *Gpx1/2* gene expression in gastric mucosa after *H. pylori* infection[39]. Association of the elevated expression of *Gpx1/2* gene with tumorigenesis could be due to its anti-apoptotic activity[40].

GSDMB encodes Gasdermin-B protein (GSDMB) the function of which is largely unknown. The expression of *GSDMB* has been associated with differentiated epithelial cells and with regions containing proliferating cells or stem cells, respectively, of the esophagus and the gastric mucosa[41,42].

JAZF1, also known as *TIP27*, encodes a transcriptional repressor of *NR2C2*, also known as *TAK1* or *TR4*[76]. Mice deficient in *NR2C2* show low IGF1 serum concentrations and perinatal and early postnatal hypoglycemia, as well as growth retardation[77]. *JAZF1* also affects variation in human height[78]. SNPs in *JAZ1F* have been associated with type II diabetes[79], prostate[80] and endometrial cancer[81] and with systemic lupus erythematosus[82]. However, the role of *JAZF1* in immune response and autoimmunity remains to be elucidated.

LSP1 encodes a leukocyte-specific protein 1 (LSP1), a Ca^{2+}-activated, intracellular

filamentous actin-binding protein that interacts with the cytoskeleton and is expressed in hematopoietic lineage and in endothelial cells[70]. Evidence from mice model studies suggest that LSP1 plays a negative regulatory role on neutrophil and T cell migration[71,72]. A recent study identified a novel *LSP1* deletion variant for RA susceptibility through CNV GWAS[73]. The copy number of *LSP1* was found to be significantly lower in RA patients and was associated with increased T cell migration[73]. We found a positive correlation of LSP1 expression (in $CD14^+$ cells) with UC, but not with CD. UC, as well as CD, is characterized by an increased infiltration of immune cells in inflamed tissues. Our finding is therefore surprising if we consider the concept of an association between increased cell migration with *LSP1* CNVs and LSP1 insufficiency. It is possible that LSP1 plays an additional, yet unknown role in monocytes. On the other hand, if LSP1 participates actively in the cross-talk between leukocytes and endothelial cells during leukocyte transmigration, the physiological differences in microvasculature and the integrins involved may dictate organ-specific roles for LSP1 in leukocyte recruitment into the inflammatory sites.

NXPE1: Encodes Neuroexophilin and PC-esterase domain family member 1 (NXPE1). A human gastointestinal tract (GIT) specific transcriptome and proteome study validate the expression pattern of this gene and protein in the intestine [74]. NXPE1 was recently identified as a novel target gene for IBD-associated variants[75]. Its function remains largely unknown.

ORMDL3 encodes ORM1-like protein 3, a negative regulator of sphingolipid synthesis and a regulator of endoplasmic reticulum-mediated calcium signaling[45]. ORMDL3 is involved in the regulation of eosinophil and T cell functios[46,47]. It also facilitate B cells survival and regulates autophagy through the ATF6 signaling pathway[48]. Genetic variants regulating ORMDL3 expression have been associated with susceptibility to ashma[49], T1D[50], atherosclerosis[51], ankylosing spondylitis[52] and IBD[53]. ORMDL3 might be associated with IBDs and other autoimmune and inflammatory diseases by activating ERS, inducing autophagy and/or promoting immune cells activation.

REXO2 encodes an oligoribonuclease protein. Its depletion, using RNAi, causes a significant decrease of mtDNA and mtRNA and impaired *de novo* mitochondrial protein synthesis[83]. REXO2's function remains unknown but it may be involved in the well documented mitochondrial defects associated with IBD[84].

RNASET2 is the only RNase T2 family member in humans and is potentially involved in the inhibition of tumorigenesis, metastasis and angiogenesis[85,86]. Loss-of-function of RNASET2 protects fibroblasts from oxidative stress[89] while its overexpression in melanocytes and keratinocytes sensitizes these cells to oxidative-stress-induced apoptosis[90]. Interestingly, CD is characterized by an impaired immune cells apoptosis associated with elevated H_2O_2 in PBMC during the active phase of the disease[91]. Although speculative, it is possible that reduced *RNASET2* contributes to the altered oxidative stress in CD.

SKAP2 encodes the Src kinase-associated phosphoprotein 2 (Skap2), a cytosolic adaptor protein expressed in a variety of cell types including hematopoietic cells[54,55,56]. Skap2 has been implicated in cell adhesion through association to integrins and cytoplasmic actin[55], and is required for global actin reorganization. It interacts with different molecules implicated in integrin signaling events[54,56,57]. Loss of Skap2 in mice results in reduced inflammation in experimental autoimmune encephalomyelitis as well as defects in macrophage migration into tumor metastasis, suggesting a physiologically important role of Skap2 for leukocyte recruitment *in vivo*[55,58].

UBE2L3 gene encodes an atypical Ubiquitin E2 Conjugase (UBE2L3) the role of which has been recently uncovered. It is an indirect human and mouse Caspase-1 target and plays an important role in the maturation of IL-1β. UBE2L3 depletion in mice increases pro-IL-1β levels and mature-IL-1β secretion by inflammasomes[61]. Several GWAS identified polymorphisms in the genomic locus of *UBE2L3* that are associated with multiple autoimmune diseases[62] including CD[29]. Decreased secretion of the inflammasome cytokine IL-1β was noted in monocytes of Crohn's disease patients[63]. It is therefore tempting to speculate that *UBE2L3* contributes to disease at least partially by modulating IL-1β secretion.

ZMIZ1 encodes Zmiz1, a member of the protein inhibitor of activated STAT (PIAS)-like family of coregulators[64]. Zmiz1 is widely and variably expressed[65]. In GWAS, a SNP within ZMIZ1 gene was associated with early-onset Crohn's disease and IBD[66]. *ZMIZ1* is co-expressed with activated *NOTCH1* across a broad range of T-ALL oncogenomic subgroups. Its inhibition slows human T-ALL cell proliferation and/or sensitizes them to γ-Secretase inhibitors (GSI)[67]. Evidence from Zmiz1-deficient mice demonstrated that Zmiz1 is a direct Notch1 cofactor that heterogeneously regulates Notch1 target genes and plays an important role in T cells development[68]. Altered expression of *ZMIZ1* has been reported to affect Smad3-mediated transcription[69]. Interestingly, our analysis shows that increased UC disease risk was associated with decrease of both *SMAD3* and *ZMIZ1* expression while no association was observed with *NOTCH1*. This association was observed in different tissues/cell types suggesting a possible trans effect of *ZMIZ1* on *SMAD3* expression.

Supplementary Table 1

Cell type	Naive (r^2 based)			Frequentist (Nica et al., 2010)			Theta-based		
	Overlaps observed	Overlaps expected	P value	Overlaps observed	Overlaps expected	P value	Overlaps observed	Overlaps expected	P value
CD4	12	3.3	< 0.01	14	4.9	< 0.01	17	8.4	< 0.01
CD8	12	3.5	< 0.01	18	4.3	< 0.01	16	6.9	< 0.01
CD14	8	3.3	0.061	9	4.7	0.211	10	7.1	0.720
CD15	4	1.9	0.646	4	2	0.720	7	5.1	0.909
CD19	7	2	0.010	7	3.6	0.410	12	5.8	0.044
PLA	4	0.9	0.010	3	0.9	0.475	5	1.8	0.119
IL	4	1.6	0.432	7	2.1	0.027	8	4.1	0.281
TR	6	2.6	0.211	5	3.5	0.928	11	6	0.086
RE	5	1.5	0.103	6	2.4	0.204	9	5.5	0.509

Enrichment of DAP-EAP matching in 63 of 97 CD risk loci covered by the Immunochip. For each cell type, we provide the number of matches (or overlaps) observed with the top disease-associated SNPs (MAF > 0.05), as well as the number of matches expected with the same number of SNPs (MAF > 0.05) sampled at random in the same 63 risk loci. The analyses were conducted using three "colocalisation" methods (Naive, Frequentists and Theta-based). The p-values were determined by simulation (1,000 sets of 63 randomly sampled SNPs) and Bonferroni corrected for the analysis of 9 cell types. < 0.01 means that the number of matches observed with the real disease-associated SNPs was never observed with any set (out of 1,000) of randomly sampled SNPs.

Supplementary Table 2

Tissue	Nr of samples	Nr of probes	Nr of PCs
CD4	303	13,466	38
CD8	294	13,317	35
CD19	282	12,648	40
CD14	286	13,170	36
CD15	289	11,069	27
PLA	251	6,565	23
IL	200	15,401	59
TR	271	15,082	50
RE	267	14,844	53

Number of usable samples, probes and PC for each tissue type.

Supplementary Table 3

Tissue	Nr of probes	FDR≤0.25	FDR≤0.10	FDR≤0.05	FDR≤0.01
CD4	13,466	7,417	4,957	4,176	3,247
CD8	13,317	6,760	4,309	3,599	2,779
CD19	12,648	4,984	3,138	2,549	1,953
CD14	13,170	7,118	4,728	3,961	3,106
CD15	11,069	3,611	2,396	1,983	1,512
PLA	6,565	1,404	996	854	653
IL	15,401	2,769	1,728	1,426	1,031
TR	15,082	5,183	3,391	2,807	2,160
RE	14,844	4,180	2,726	2,295	1,731

Number of cis-eQTL found in the nine analyzed cell types for different FDR thresholds (see also Suppl. Figure 7).

Supplementary Figures

1. Supplementary Figure 1

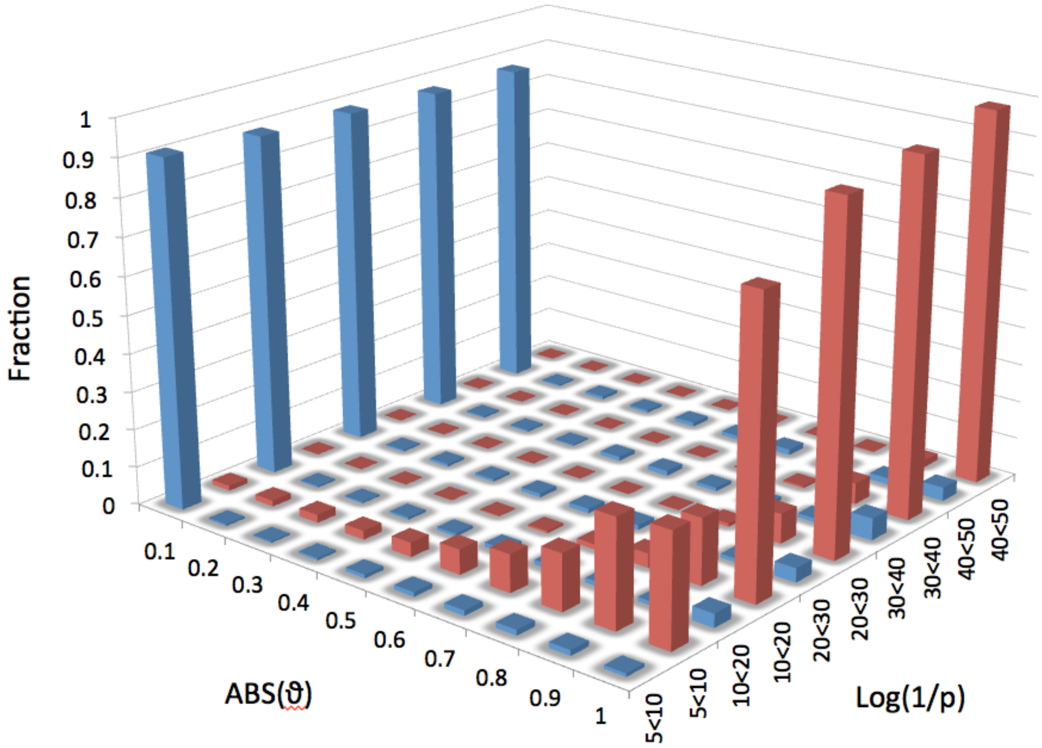

Absolute values of ϑ for pairs of eQTL driven by distinct regulatory variants (blue), and for pairs of eQTL driven by the same regulatory variants (red). The first (blue) were obtained by confronting real cis-eQTL with in silico simulated eQTL explaining the same variance as the real eQTL but driven by a randomly chosen SNPs in a 2Mb window centered around the probe. The second (red) were obtained by confronting eQTL obtained by reanalyzing two mutually exclusive halves of the CEDAR population separately in a region harboring a real cis-eQTL. It can be seen that ϑ very effectively discriminates between pairs of eQTL driven by distinct (blue) vs the same (red) regulatory variants. By choosing 0.6 as threshold value for ϑ, one captures most red pairs (~88%) with minimum contamination of blue pairs (~5%). Log(1/p): eQTL are sorted by the smallest log(1/p) value of the two eQTL being compared.

2. Supplementary Figure 2

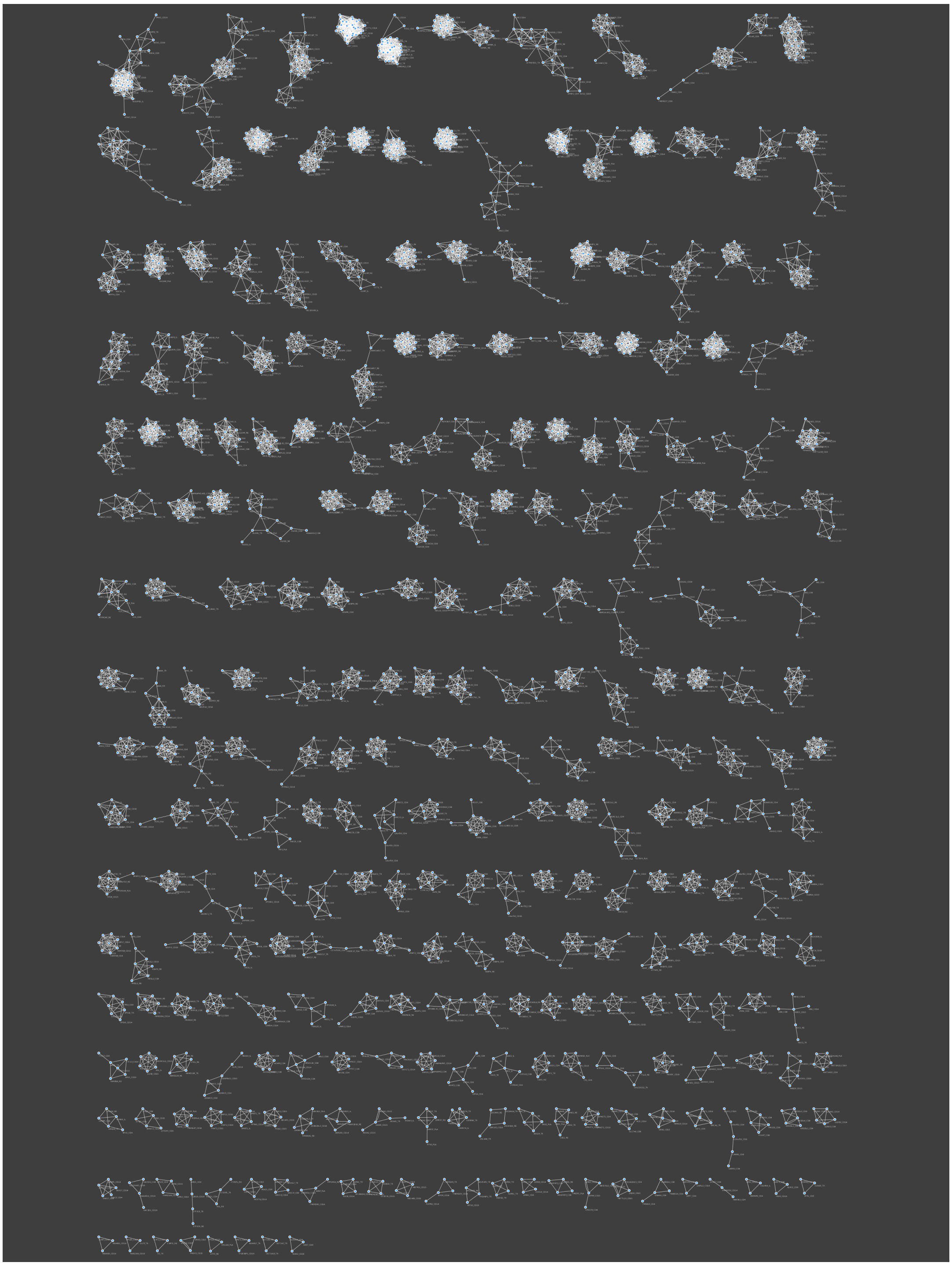

Graphical representation (using Cytoscape[1]) of 269 cis acting regulatory modules (cRM) including at least three genes (see Suppl. Table 2). Every node corresponds to a cis-eQTL involving a specific gene-tissue combination. Edges connect pairs of cis-eQTL for which $|\vartheta| \geq 0.6$.

1. Shannon, P. et al. Genome Res. 13, 2498-2504 (2003).

3. Supplementary Figure 3

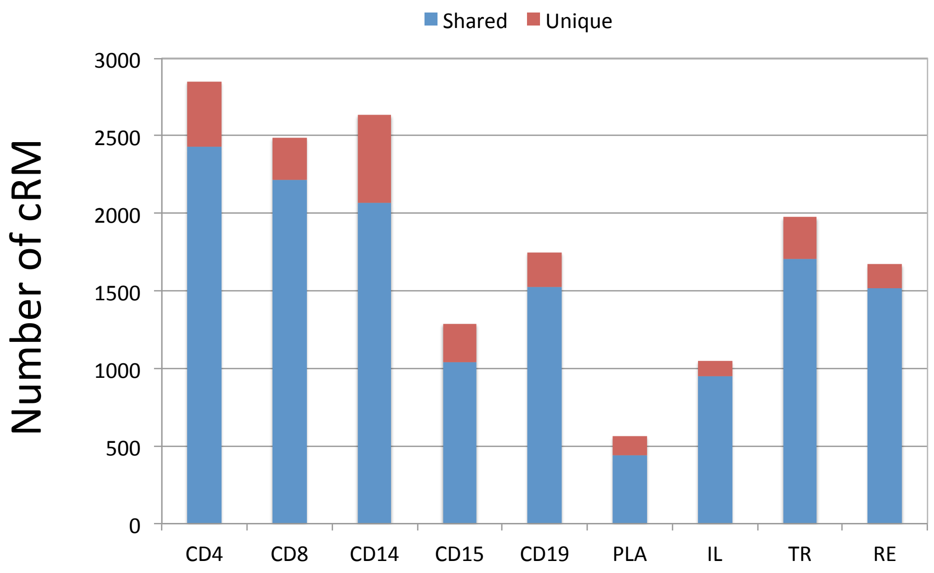

Number of cRM detected in each cell type. Blue: shared cRM (i.e. also detected in at least one other cell type). Red: Unique (i.e. detected only in that cell type).

4. Supplementary Figure 4

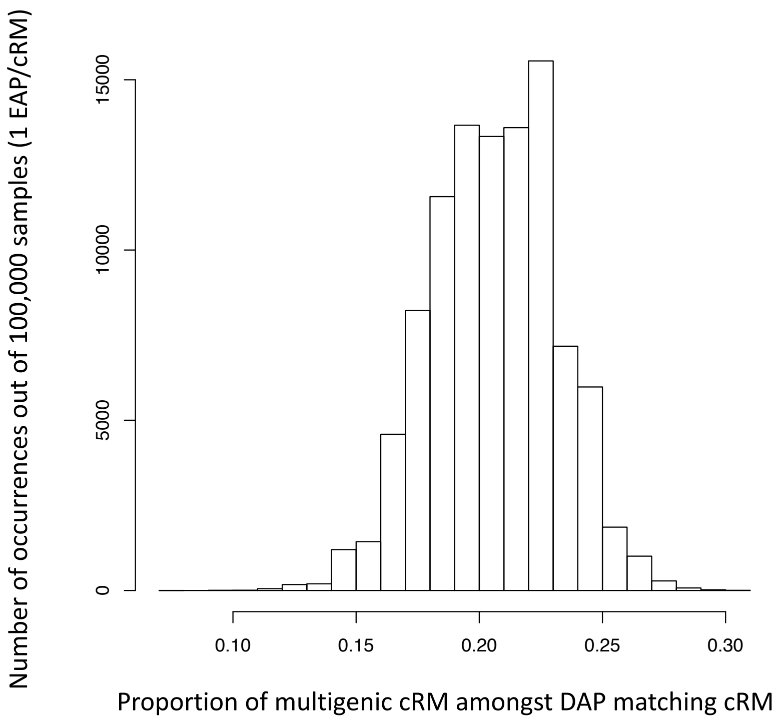

Across the entire genome, the proportion of multigenic cRM was shown to be 0.10 (see also main text, figure 1B). Amongst DAP matching cRM (mapping to 63 of 200 studied IBD risk loci; main text Table 1) this proportion was shown to be 0.33, hence a highly significant enrichment. To ensure that this enrichment was not only due to the fact that matching between DAP and EAP was de facto tested multiple times for multigenic cRM and only once for other cRM, we only tested one randomly sampled EAP per cRM (whether monogenic or multigenic). This was repeated 100,000 times and yielded the distribution of the proportion of multigenic cRM amongst DAP matching cRM shown above. The average was 0.22, and we never observed values ≤ 0.11, i.e. the genome-wide average.

5. Supplementary Figure 5

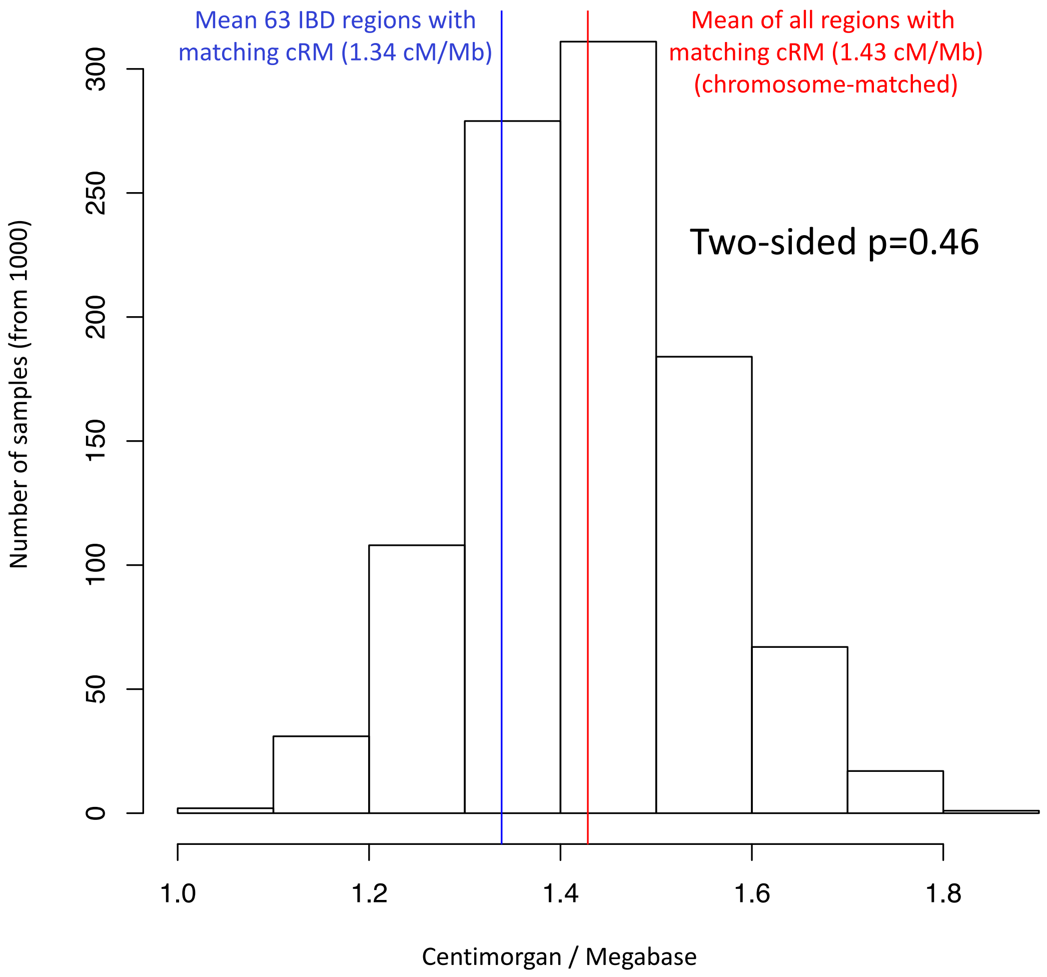

The 63 IBD risk loci with matching cRM are 2- to 3-fold enriched in multigenic cRM ($p \leq 10^{-5}$). This could be due to the fact that the LD is higher in IBD regions than in the rest of the genome. To test this, we downloaded LD-based recombination maps of the human genome from https://github.com/joepickrell/1000-genomes-genetic-maps. The average recombination rate across the human genome was 1.23 centimorgan per megabase (cM/Mb). The average recombination rate for the 63 IBD risk loci with matching cRM was 1.34 cM/Mb, i.e. less LD than in the rest of the genome. Regions encompassing eQTL (and hence cRM) may differ from the rest of the genome with regards to LD. Thus, we further sampled 1,000 sets of 63 loci centered on cRM (from our list of 9,720) that were matched for size and chromosomal location with the 63 cRM-matching IBD risk loci. The mean recombination rate for the cRM-centered genome was 1.43 cM/Mb. The figure shows the frequency distribution of the corresponding mean cRM/Mb per set (black), the mean of means of the 1,000 sets of 63 randomly drawn loci (red), and the mean of the 3 IBD risk loci (blue). The mean of the 63 IBD risk loci did not differ significantly from the rest of the cRM centered portion of the genome (two-tailed p-value: 0.46).

6. Supplemental Figure 6

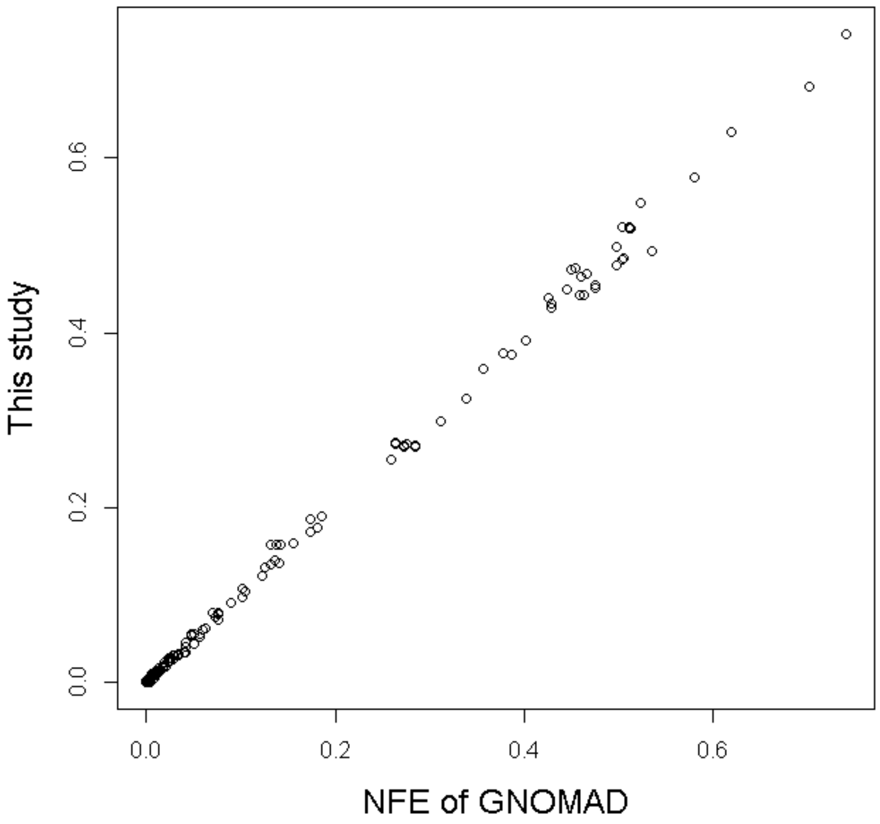

Comparison of the alternative allele frequency for 1,781 variants observed in this study and in 55,860 non-Finnish European samples from the GNMAD study.

7. Supplementary Figure 7

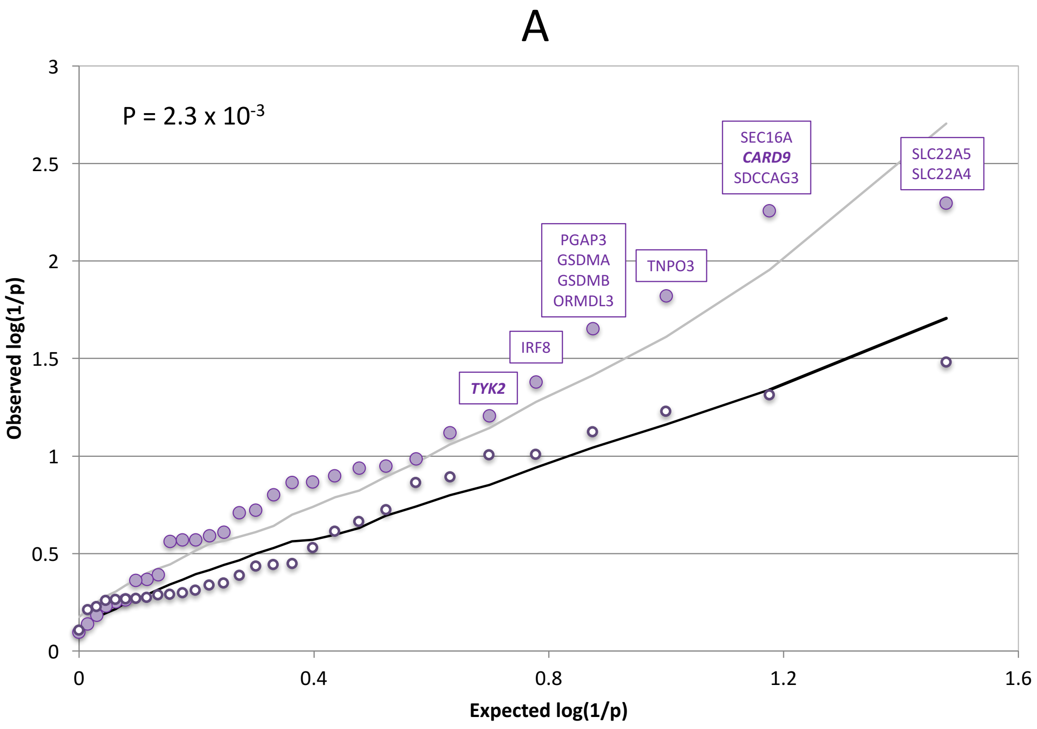

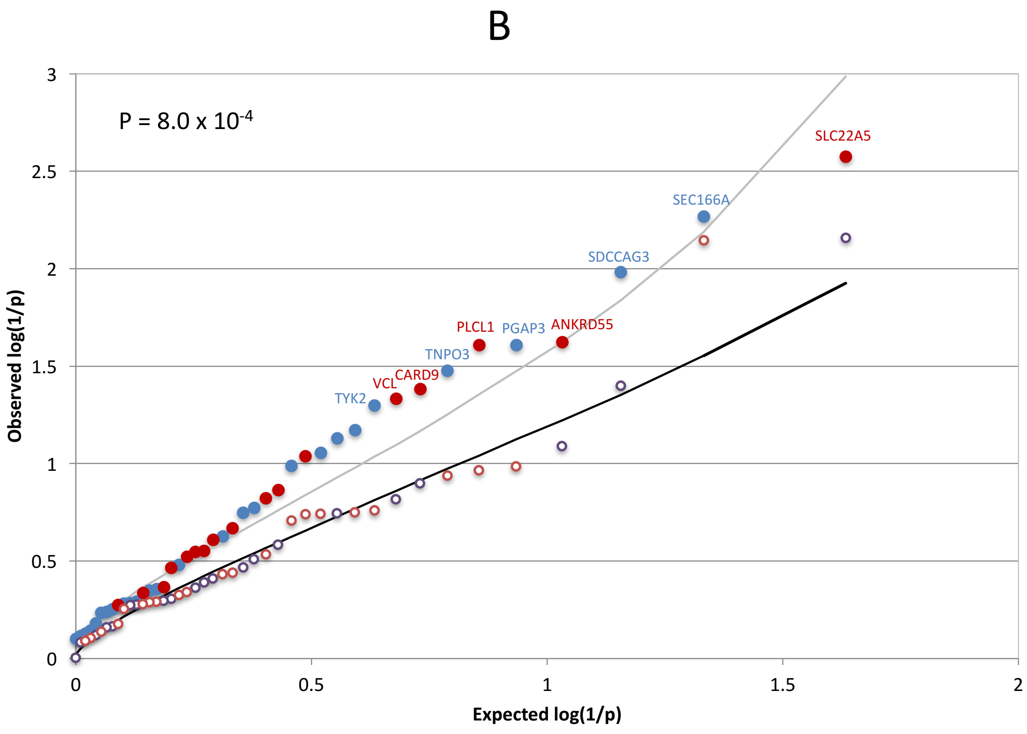

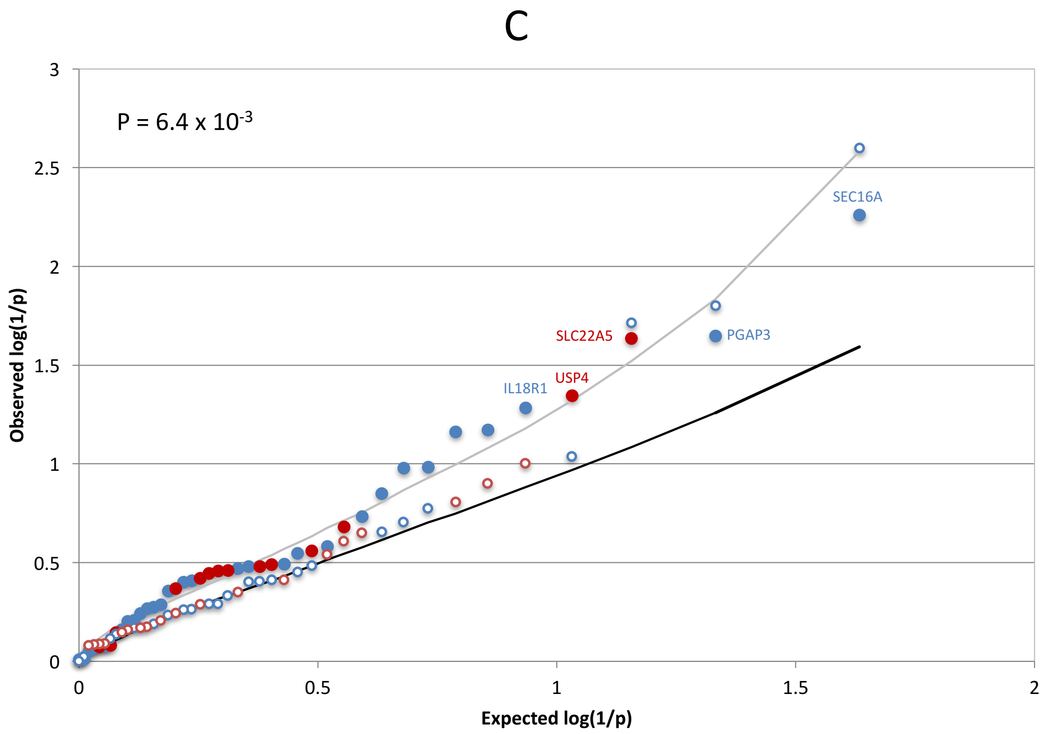

QQ-plot for the module-based burden test (**A**), disease plus age-of-onset-based burden test (**B**), and disease plus familiality-based burden test (**C**). Ranked log(1/p) values obtained when considering LoF and damaging variants (full circles), or synonymous variants (empty circles). The circles are labeled in blue when the best p-value for that gene is obtained with CAST, in red when the best p-value is obtained with SKAT, or in purple for the module-based test (as some genes in the module may have their best p-value with CAST and other with SKAT). The black line corresponds to the median log(1/p) value obtained (for the corresponding rank) using the same approach on permuted data (LoF and damaging variants). The grey line marks the upper limit of the 95% confidence band. The name of the genes/modules exceeding the nominal p-value of 0.05 are given. The inset p-values correspond to the significance of the upwards shift in log(1/p) values estimated by permutation.

8. Supplementary Figure 8

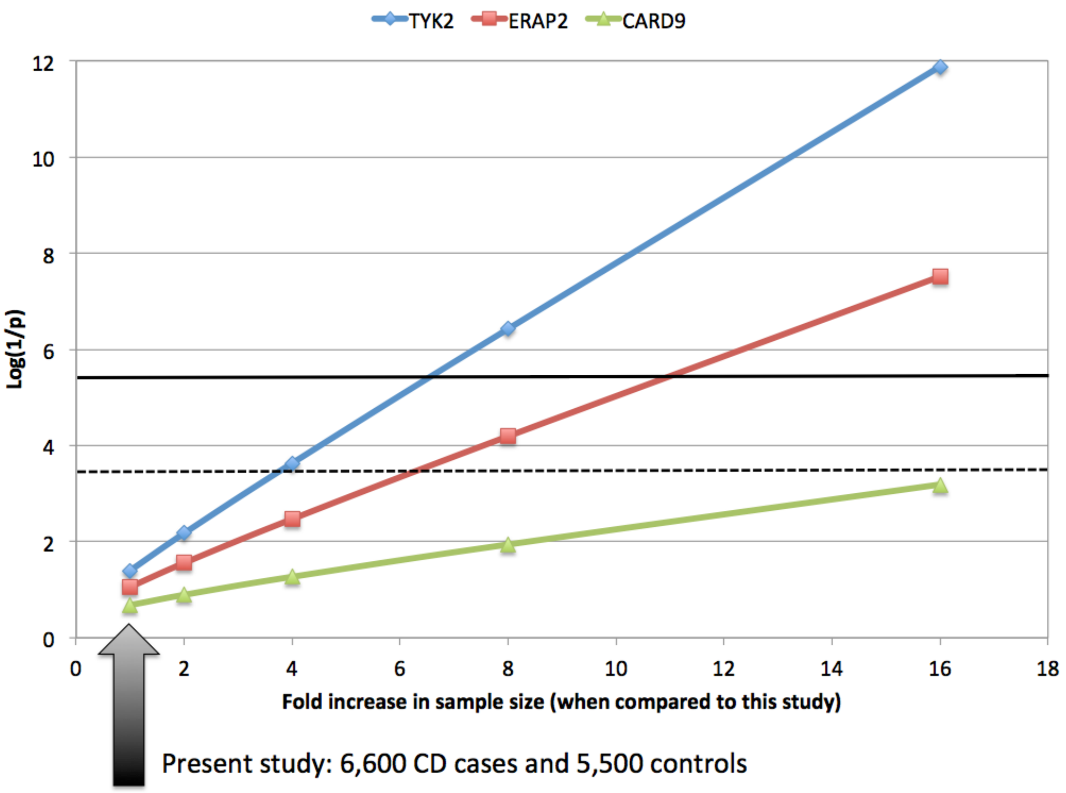

Effect of increasing sample size on the *log(1/p)* values of a one-sided burden test assuming that the effects observed for *TYK2* (blue), *ERAP2* (red) and *CARD9* (green) observed in this study are real unbiased. The dotted horizontal black line corresponds to an hypothetical experiment-wide significance threshold assuming the realization of 200 independent tests (targeting for instance 100-200 genes selected on the basis of coincident DAP-EAP patterns). The plain horizontal black line corresponds to an hypothetical genome-wide significance threshold assuming the realization of 20,000 independent tests (targeting all genes). It can be seen that an at least 4-fold increase in sample size is needed to achieve significance in the first scenario and at least 7-fold increase in the second scenario.

9. Supplementary Figure 9

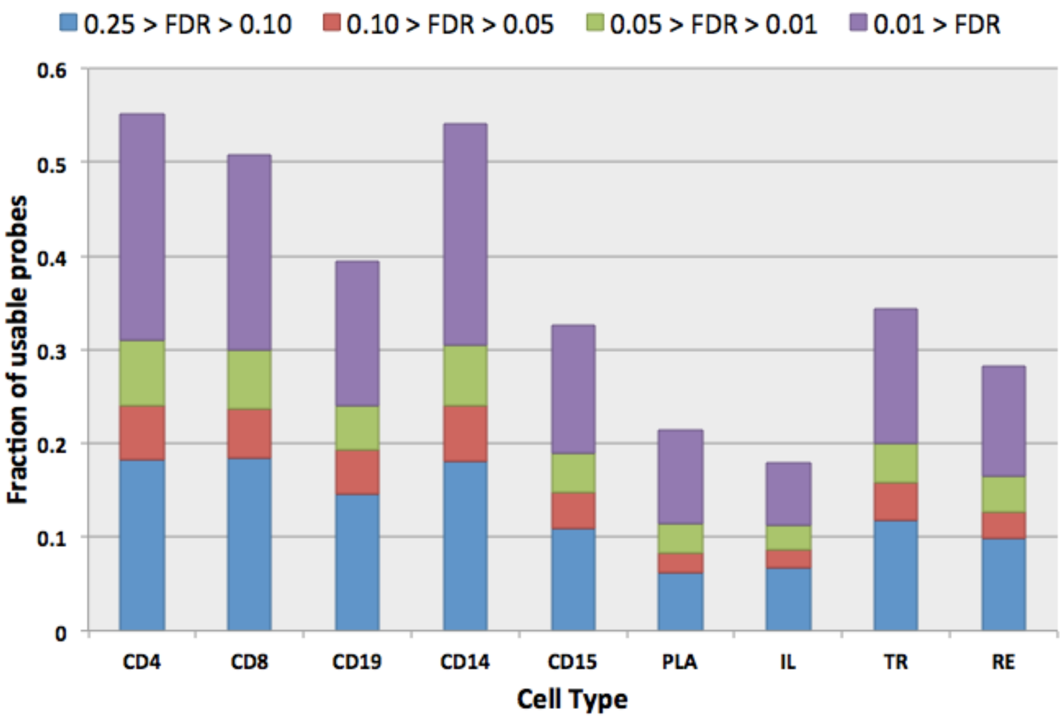

Proportion of usable probes with cis-eQTL at various levels of FDR in the nine analyzed cell types.

10. Supplementary Figure 10

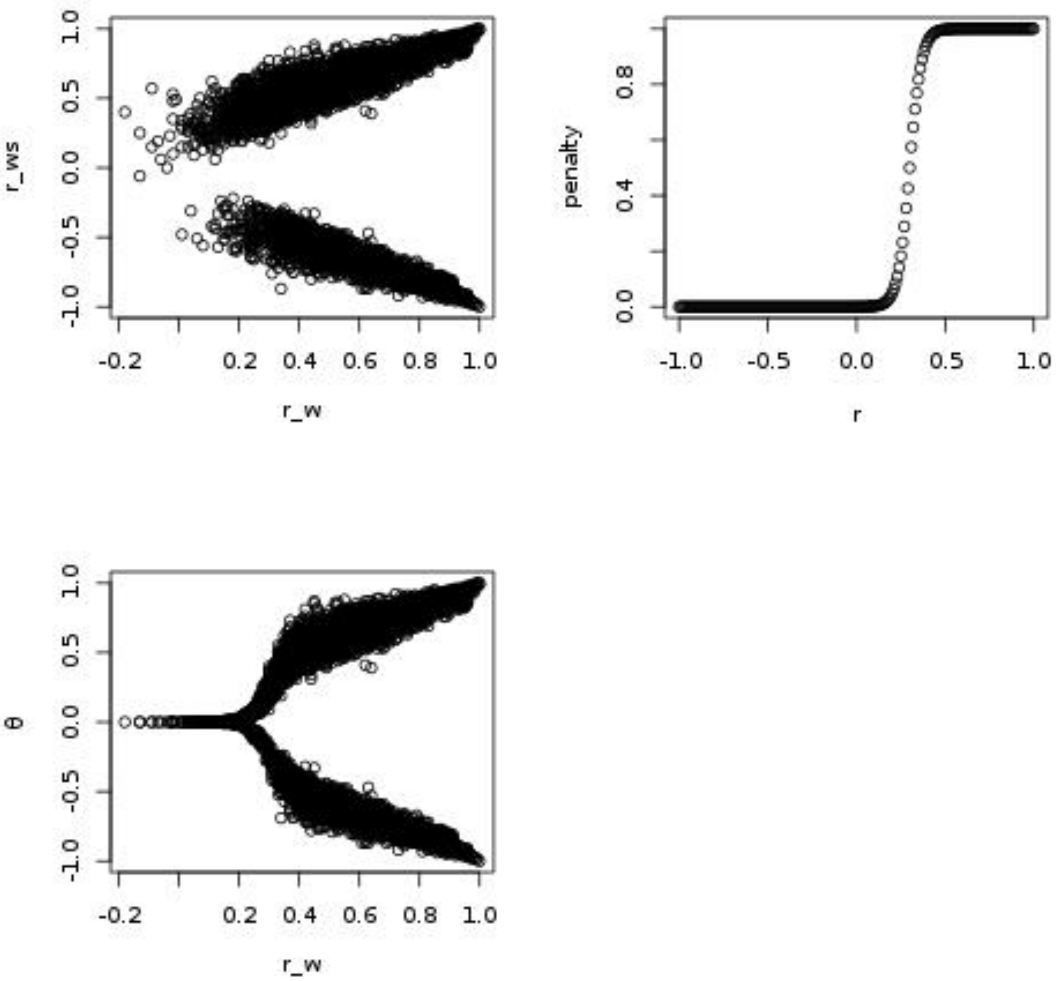

Graphical illustration of the relationship between r_w, r_{ws} and ϑ. The penalty function applied to r_{ws} to generate ϑ, corresponds to $\frac{1}{1+e^{-k(r_w-T)}}$.. The graph is shown for k = 30 and T=0.3, the values used in this study.

The point here is that if two association patterns are "similar" (driven by the same variants), the correlation (r_w in Suppl. Methods) between –log(1/p) values is expected to be positive. If two association patterns are different (driven by distinct variants) they may generate strong <u>negative</u> correlations (r_w). The first part of the method aims at weeding out such instances (negative r_w). One way to do this is to choose a simple threshold value for r_w. We herein propose an approach that offers more flexibility: it generates a penalty that increases when the correlation decreases with an adaptable rate. As shown in Suppl. Fig. 8, the values of k=30 and T=0.3 essentially correspond to a threshold value of 0.3. As can also be seen from Suppl. Fig. 8, there is (as expected) a strong linear relationship with slope 1 between r_w and $|r_{ws}|$ (and hence between r_w and $|\vartheta|$ for pairs with $r_w > 0.3$). Because we subsequently use a threshold value $|\vartheta| \geq 0.6$, the choice T has very little impact on the outcome unless one approaches 0.6.

11. Supplementary Figure 11

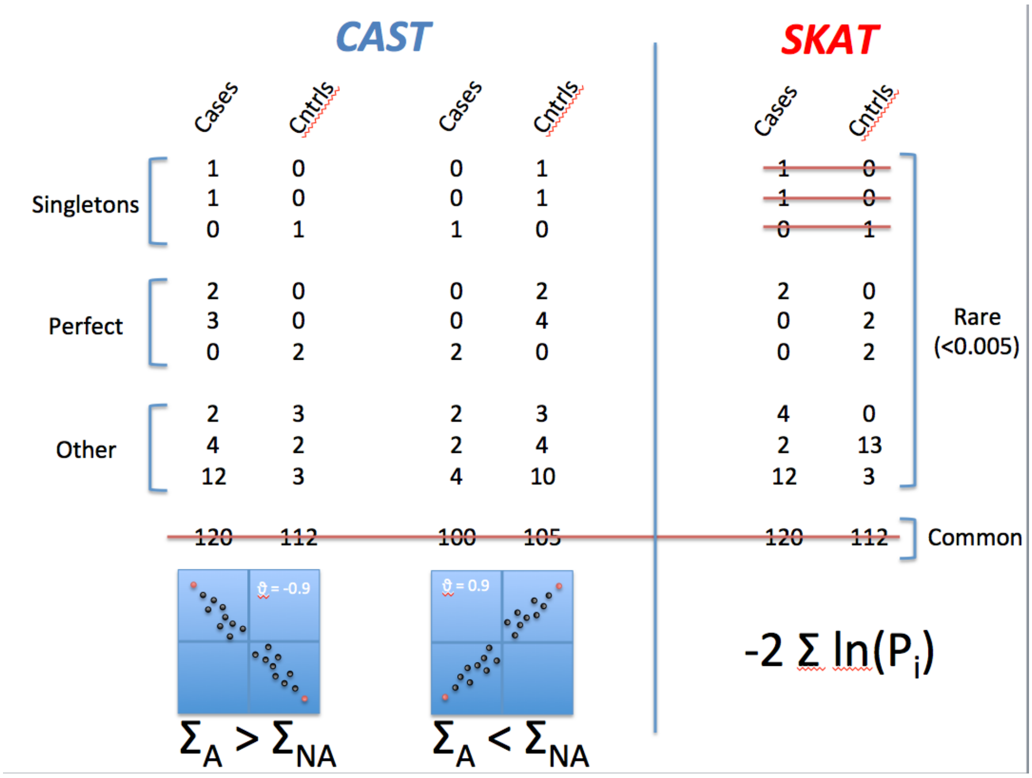

Schematic representation of the key features of the implemented “burden test”.

The analysis is restricted to rare variants with MAF < 0.005 to ensure that the new signal is independent of the one that lead to the identification of the corresponding risk loci by GWAS (based on common and low frequency variants). Variants can be sorted in (i) singletons (i.e. observed only ones in the analyzed samples), (ii) perfect (i.e. observed more than ones in the sample but perfectly associated with disease status), and (iii) other (i.e. observed more than ones in the sample in both cases and controls).

We test two hypotheses. The first assumes that disruptive variants are either enriched in cases or in controls as a function of the sign of the correlation between DAP and EAP (if decreased expression is associated with increased risk, disruptive “risk” variants are expected to be enriched in cases; if increased expression is associated with increased risk, disruptive “protective” variants are expected to be enriched in controls). The test is implemented with CAST and in essence performs a one-sided test of independence (what is the probability to observe the excess of disruptive variants in cases (respectively controls) by chance alone?). The second hypothesis tests whether the distribution of the variants in cases and controls is characterized by too many variants that tend to be overrepresented either in cases or in controls. Thus, this hypothesis allows some disruptive variants to increase risk and others to be protective. This hypothesis does not use information from singletons. Testing this hypothesis is implemented with SKAT. It can be seen in simplified form as combining the p-values (from a test of independence) across variants (without considering the sign of the effect) using for instance Fisher’s method.

12. Supplementary Figure 12

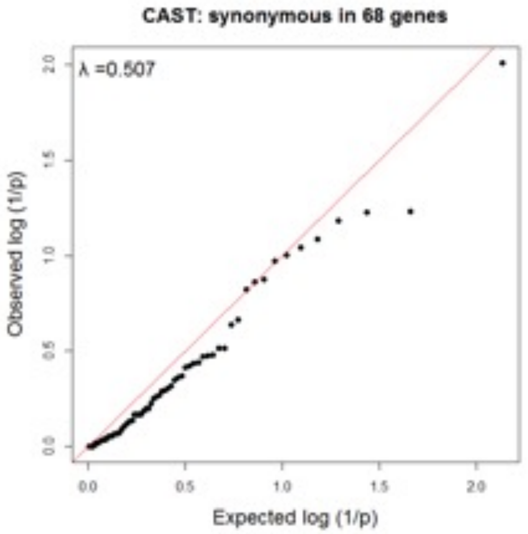

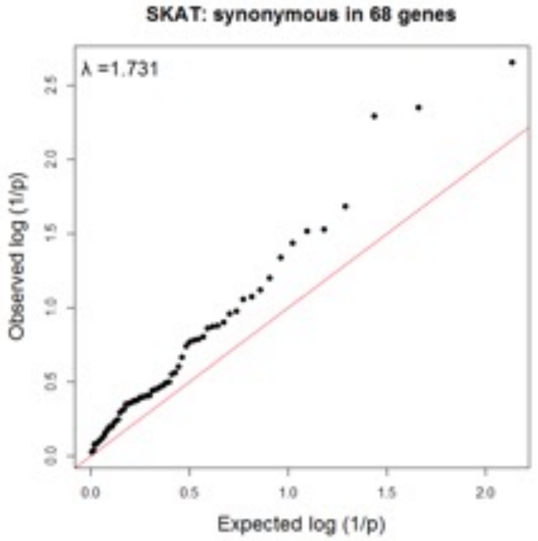

Distribution of permutation-based –log(p) values obtained for 68 analyzed genes with synonymous variants using CAST (A), and SKAT (B), indicating that CAST is conservative (, while SKAT is too permissive ($\lambda_{GC} = 1.73$). The 68 genes correspond to the 47 genes reported in this study, plus 21 genes sequenced in the same cohort as part of another study.

1. Dinarello, C. A., Novick, D., Kim, S. & Kaplanski, G. Interleukin-18 and IL-18 binding protein. *Frontiers in Immunology* **4,** (2013).
2. Elinav, E. *et al.* NLRP6 inflammasome regulates colonic microbial ecology and risk for colitis. *Cell* **145,** 745–757 (2011).
3. Salcedo, R. *et al.* MyD88-mediated signaling prevents development of adenocarcinomas of the colon: role of interleukin 18. *J. Exp. Med.* **207,** 1625–36 (2010).
4. Nowarski, R. *et al.* Epithelial IL-18 Equilibrium Controls Barrier Function in Colitis. *Cell* **163,** 1444–1456 (2015).
5. Dinarello, C. A. Interleukin-18 and the Pathogenesis of Inflammatory Diseases. *Semin. Nephrol.* **27,** 98–114 (2007).
6. Heinrich, P. C. *et al.* Principles of interleukin (IL)-6-type cytokine signalling and its regulation. *Biochem. J.* **374,** 1–20 (2003).
7. Mitsuyama, K. *et al.* Therapeutic strategies for targeting the IL-6/STAT3 cytokine signaling pathway in inflammatory bowel disease. *Anticancer Res.* **27,** 3749–56
8. Atreya, R. *et al.* Blockade of interleukin 6 trans signaling suppresses T-cell resistance against apoptosis in chronic intestinal inflammation: evidence in Crohn disease and experimental colitis in vivo. *Nat. Med.* **6,** 583–588 (2000).
9. Ito, H. *et al.* A Pilot Randomized Trial of a Human Anti-Interleukin-6 Receptor Monoclonal Antibody in Active Crohn's Disease. *Gastroenterology* **126,** 989–996 (2004).
10. Jones, S. A., Scheller, J. & Rose-John, S. Therapeutic strategies for the clinical blockade of IL-6/gp130 signaling. *J. Clin. Invest.* **121,** 3375–3383 (2011).
11. Lesourne, R. *et al.* Themis, a T cell-specific protein important for late thymocyte development. *Nat. Immunol.* **10,** 840–7 (2009).
12. Fu, G. *et al.* Themis controls thymocyte selection through regulation of T cell antigen receptor-mediated signaling. *Nat. Immunol.* **10,** 848–56 (2009).
13. Neurath, M. F. Cytokines in inflammatory bowel disease. *Nat. Rev. Immunol.* **14,**

329–342 (2014).

14. Palumbo, R. *et al.* APEH Inhibition Affects Osteosarcoma Cell Viability via Downregulation of the Proteasome. *Int. J. Mol. Sci.* **17,** 1614 (2016).

15. Cleynen, I. *et al.* Genetic and microbial factors modulating the ubiquitin proteasome system in inflammatory bowel disease. *Gut* **63,** 1265–74 (2014).

16. Li, J., Mahajan, A. & Tsai, M.-D. Ankyrin Repeat: A Unique Motif Mediating Protein−Protein Interactions †. *Biochemistry* **45,** 15168–15178 (2006).

17. International Multiple Sclerosis Genetics Consortium (IMSGC) *et al.* Analysis of immune-related loci identifies 48 new susceptibility variants for multiple sclerosis. *Nat. Genet.* **45,** 1353–60 (2013).

18. Viatte, S. *et al.* Genetic markers of rheumatoid arthritis susceptibility in anti-citrullinated peptide antibody negative patients. *Ann. Rheum. Dis.* **71,** 1984–90 (2012).

19. Wiley, S. E., Murphy, A. N., Ross, S. A., van der Geer, P. & Dixon, J. E. MitoNEET is an iron-containing outer mitochondrial membrane protein that regulates oxidative capacity. *Proc. Natl. Acad. Sci. U. S. A.* **104,** 5318–5323 (2007).

20. Geldenhuys, W. J., Leeper, T. C. & Carroll, R. T. mitoNEET as a novel drug target for mitochondrial dysfunction. *Drug Discovery Today* **19,** 1601–1606 (2014).

21. Goldberg, N. D. Iron deficiency anemia in patients with inflammatory bowel disease. *Clinical and Experimental Gastroenterology* **6,** 61–70 (2013).

22. Kojima, S., Sher-Chen, E. L. & Green, C. B. Circadian control of mRNA polyadenylation dynamics regulates rhythmic protein expression. *Genes Dev.* **26,** 2724–2736 (2012).

23. Maillo, C. *et al.* Circadian- and UPR-dependent control of CPEB4 mediates a translational response to counteract hepatic steatosis under ER stress. *Nat. Cell Biol.* **19,** 94–105 (2017).

24. Watabe-Uchida, M., John, K. A., Janas, J. A., Newey, S. E. & Van Aelst, L. The Rac Activator DOCK7 Regulates Neuronal Polarity through Local Phosphorylation of Stathmin/Op18. *Neuron* **51,** 727–739 (2006).

25. Blasius, A. L. *et al.* Mice with mutations of Dock7 have generalized hypopigmentation and white-spotting but show normal neurological function. *Proc. Natl. Acad. Sci.* **106,** 2706–2711 (2009).

26. Zhang, Z. *et al.* Functional interaction of ERAP2 and HLA-B27 activates the unfolded protein response. *Arthritis Rheumatol.* (2016). doi:10.1002/art.40033

27. Andrés, A. M. *et al.* Balancing selection maintains a form of ERAP2 that undergoes nonsense-mediated decay and affects antigen presentation. *PLoS Genet.* **6,** 1–13 (2010).

28. Saveanu, L. *et al.* Concerted peptide trimming by human ERAP1 and ERAP2 aminopeptidase complexes in the endoplasmic reticulum. *Nat. Immunol.* **6,** 689–97 (2005).

29. Franke, A. *et al.* Genome-wide meta-analysis increases to 71 the number of confirmed Crohn's disease susceptibility loci. *Nat. Genet.* **42,** 1118–25 (2010).

30. Damjanovich, L., Volkó, J., Forgács, A., Hohenberger, W. & Bene, L. Crohn's disease alters MHC-rafts in CD4 + T-cells. *Cytom. Part A* **81 A,** 149–164 (2012).

31. Dodane, V. & Kachar, B. Identification of isoforms of G proteins and PKC that colocalize with tight junctions. *J. Membr. Biol.* **149,** 199–209 (1996).

32. Meyer, T. N., Schwesinger, C. & Denker, B. M. Zonula occludens-1 is a scaffolding protein for signaling molecules: G??12 directly binds to the Src homology 3 domain and regulates paracellular permeability in epithelial cells. *J. Biol. Chem.* **277,** 24855–24858 (2002).

33. Sabath, E. *et al.* Gα12 regulates protein interactions within the MDCK cell tight junction and inhibits tight-junction assembly. *J. Cell Sci.* **121,** 814–824 (2008).

34. Fukuhara, S., Chikumi, H. & Gutkind, J. S. RGS-containing RhoGEFs: the missing link between transforming G proteins and Rho? *Oncogene* **20,** 1661–1668 (2001).

35. Landy, J. *et al.* Tight junctions in inflammatory bowel diseases and inflammatory bowel disease associated colorectal cancer. *World Journal of Gastroenterology* **22,** 3117–3126 (2016).

36. Herroeder, S. *et al.* Guanine Nucleotide-Binding Proteins of the G12 Family Shape Immune Functions by Controlling CD4+ T Cell Adhesiveness and

Motility. *Immunity* **30,** 708–720 (2009).

37. Pavlick, K. P. *et al.* Role of reactive metabolites of oxygen and nitrogen in inflammatory bowel disease 1,2 1This article is part of a series of reviews on 'Reactive Oxygen and Nitrogen in Inflammation.' The full list of papers may be found on the homepage of the journal. 2Gue. *Free Radic. Biol. Med.* **33,** 311–322 (2002).

38. Chu, F. F. *et al.* Bacteria-Induced Intestinal Cancer in Mice with Disrupted Gpx1 and Gpx2 Genes. *Cancer Res.* **64,** 962–968 (2004).

39. Maeda, S. *et al.* cDNA microarray analysis of Helicobacter pylori-mediated alteration of gene expression in gastric cancer cells. *Biochem. Biophys. Res. Commun.* **284,** 443–9 (2001).

40. Hockenbery, D. M., Oltvai, Z. N., Yin, X. M., Milliman, C. L. & Korsmeyer, S. J. Bcl-2 functions in an antioxidant pathway to prevent apoptosis. *Cell* **75,** 241–51 (1993).

41. Saeki, N. *et al.* GASDERMIN, suppressed frequently in gastric cancer, is a target of LMO1 in TGF-beta-dependent apoptotic signalling. *Oncogene* **26,** 6488–98 (2007).

42. Saeki, N. *et al.* Distinctive expression and function of four GSDM family genes (GSDMA-D) in normal and malignant upper gastrointestinal epithelium. *Genes, Chromosom. Cancer* **48,** 261–271 (2009).

43. Pal, L. R. & Moult, J. Genetic basis of common human disease: Insight into the role of missense SNPs from genome-wide association studies. *J. Mol. Biol.* **427,** 2271–2289 (2015).

44. Jostins, L. *et al.* Host-microbe interactions have shaped the genetic architecture of inflammatory bowel disease. *Nature* **491,** 119–24 (2012).

45. Breslow, D. K. *et al.* Orm family proteins mediate sphingolipid homeostasis. *Nature* **463,** 1048–1053 (2010).

46. Ha, S. G. *et al.* ORMDL3 promotes eosinophil trafficking and activation via regulation of integrins and CD48. *Nat. Commun.* **4,** 2479 (2013).

47. Schmiedel, B. J. *et al.* 17q21 asthma-risk variants switch CTCF binding and regulate IL-2 production by T cells. *Nat. Commun.* **7,** 13426 (2016).

48. Dang, J. *et al.* ORMDL3 Facilitates the Survival of Splenic B Cells via an ATF6α-Endoplasmic Reticulum Stress-Beclin1 Autophagy Regulatory Pathway. *J. Immunol.* (2017). doi:10.4049/jimmunol.1602124

49. Zhai, W. H., Song, C. Y., Huang, Z. G. & Sha, H. Correlation between the genetic polymorphism of ORMDL3 gene and asthma risk: a meta-analysis. *Genet. Mol. Res.* **14,** 7101–12 (2015).

50. Saleh, N. M. *et al.* Genetic association analyses of atopic illness and proinflammatory cytokine genes with type 1 diabetes. *Diabetes. Metab. Res. Rev.* **27,** 838–43 (2011).

51. Ma, X. *et al.* ORMDL3 contributes to the risk of atherosclerosis in Chinese Han population and mediates oxidized low-density lipoprotein-induced autophagy in endothelial cells. *Sci. Rep.* **5,** 17194 (2015).

52. Laukens, D. *et al.* Evidence for significant overlap between common risk variants for Crohn's disease and ankylosing spondylitis. *PLoS One* **5,** e13795 (2010).

53. McGovern, D. P. B. *et al.* Genome-wide association identifies multiple ulcerative colitis susceptibility loci. *Nat. Genet.* **42,** 332–7 (2010).

54. Asazuma, N. *et al.* Interaction of linker for activation of T cells with multiple adapter protiens in platelets activated by the glycoprotein VI-selective ligand, convulxin. *J. Biol. Chem.* **275,** 33427–33434 (2000).

55. Togni, M. *et al.* Regulation of In Vitro and In Vivo Immune Functions by the Cytosolic Adaptor Protein SKAP-HOM. *Mol. Cell. Biol.* **25,** 8052–8063 (2005).

56. Alenghat, F. J. *et al.* Macrophages require Skap2 and Sirpα for integrin-stimulated cytoskeletal rearrangement. *J. Cell Sci.* **125,** 5535–5545 (2012).

57. Königsberger, S. *et al.* HPK1 associates with SKAP-HOM to negatively regulate Rap1-mediated B-lymphocyte adhesion. *PLoS One* **5,** 1–9 (2010).

58. Tanaka, M. *et al.* SKAP2 Promotes Podosome Formation to Facilitate Tumor-Associated Macrophage Infiltration and Metastatic Progression. *Cancer Res.* **76,** 358–369 (2016).

59. Sartor, R. B. Mechanisms of disease: pathogenesis of Crohn's disease and ulcerative colitis. *Nat. Clin. Pract. Gastroenterol. Hepatol.* **3,** 390–407 (2006).

60. Ghosh, S. *et al.* Natalizumab for active Crohn's disease. *N Engl J Med* **348,** 24–32. (2003).

61. Eldridge MJ, Sanchez-Garrido J, Hoben GF, Goddard PJ, S. A. The Atypical Ubiquitin E2 Conjugase UBE2L3 Is an Indirect Caspase-1 Target and Controls IL-1β Secretion by Inflammasomes. *Cell Rep.* **18,** 1285–1297 (2017).

62. Alpi, A. F., Chaugule, V. & Walden, H. Mechanism and disease association of E2-conjugating enzymes: lessons from UBE2T and UBE2L3. *Biochem. J.* **473,** 3401–3419 (2016).

63. Lamkanfi, M., Walle, L. Vande & Kanneganti, T. D. Deregulated inflammasome signaling in disease. *Immunological Reviews* **243,** 163–173 (2011).

64. Shuai, K. & Liu, B. Regulation of gene-activation pathways by PIAS proteins in the immune system. *Nat. Rev. Immunol.* **5,** 593–605 (2005).

65. Sharma, M. *et al.* hZimp10 is an androgen receptor co-activator and forms a complex with SUMO-1 at replication foci. *EMBO J.* **22,** 6101–6114 (2003).

66. Imielinski, M. *et al.* Common variants at five new loci associated with early-onset inflammatory bowel disease. *Nat. Genet.* **41,** 1335–40 (2009).

67. Rakowski, L. A. *et al.* Convergence of the ZMIZ1 and NOTCH1 Pathways at C-MYC in Acute T Lymphoblastic Leukemias. *Cancer Res.* **73,** 930–941 (2013).

68. Pinnell, N. *et al.* The PIAS-like Coactivator Zmiz1 Is a Direct and Selective Cofactor of Notch1 in T Cell Development and Leukemia. *Immunity* **43,** 870–883 (2015).

69. Li, X., Thyssen, G., Beliakoff, J. & Sun, Z. The Novel PIAS-like Protein hZimp10 Enhances Smad Transcriptional Activity. *J. Biol. Chem.* **281,** 23748–23756 (2006).

70. Jongstra-Bilen, J. & Jongstra, J. Leukocyte-specific protein 1 (LSP1): a regulator of leukocyte emigration in inflammation. *Immunol. Res.* **35,** 65–74 (2006).

71. Wang, C. *et al.* Modulation of Mac-1 (CD11b/CD18)-Mediated Adhesion by the Leukocyte-Specific Protein 1 Is Key to Its Role in Neutrophil Polarization and Chemotaxis. *J. Immunol.* **169,** 415–423 (2002).

72. Wang, J. *et al.* Accelerated wound healing in leukocyte-specific, protein 1-deficient mouse is associated with increased infiltration of leukocytes and

fibrocytes. *J. Leukoc. Biol.* **82,** 1554–63 (2007).

73. Hwang, S.-H. *et al.* Leukocyte-specific protein 1 regulates T-cell migration in rheumatoid arthritis. *Proc. Natl. Acad. Sci.* **112,** E6535–E6543 (2015).

74. Gremel, G. *et al.* The human gastrointestinal tract-specific transcriptome and proteome as defined by RNA sequencing and antibody-based profiling. *J. Gastroenterol.* **50,** 46–57 (2015).

75. Hulur, I. *et al.* Enrichment of inflammatory bowel disease and colorectal cancer risk variants in colon expression quantitative trait loci. *BMC Genomics* **16,** 138 (2015).

76. Nakajima, T. TIP27: a novel repressor of the nuclear orphan receptor TAK1/TR4. *Nucleic Acids Res.* **32,** 4194–4204 (2004).

77. Collins, L. L. *et al.* Growth retardation and abnormal maternal behavior in mice lacking testicular orphan nuclear receptor 4. *Proc. Natl. Acad. Sci. U. S. A.* **101,** 15058–63 (2004).

78. Johansson, ??sa *et al.* Common variants in the JAZF1 gene associated with height identified by linkage and genome-wide association analysis. *Hum. Mol. Genet.* **18,** 373–380 (2009).

79. Cooper, J. D. *et al.* Meta-analysis of genome-wide association study data identifies additional type 1 diabetes risk loci. *Nat. Genet.* **40,** 1399–401 (2008).

80. Thomas, G. *et al.* Multiple loci identified in a genome-wide association study of prostate cancer. *Nat. Genet.* **40,** 310–5 (2008).

81. Koontz, J. I. *et al.* Frequent fusion of the JAZF1 and JJAZ1 genes in endometrial stromal tumors. *Proc. Natl. Acad. Sci.* **98,** 6348–6353 (2001).

82. Martin, J. E. *et al.* A systemic sclerosis and systemic lupus erythematosus pan-meta-GWAS reveals new shared susceptibility loci. *Hum. Mol. Genet.* **22,** 4021–4029 (2013).

83. Bruni, F., Gramegna, P., Oliveira, J. M. A., Lightowlers, R. N. & Chrzanowska-Lightowlers, Z. M. A. REXO2 is an oligoribonuclease active in human mitochondria. *PLoS One* **8,** e64670 (2013).

84. Matondo, A. & Kim, S. S. Targeted-mitochondria antioxidants therapeutic implications in inflammatory bowel disease. *J. Drug Target.* 1–8 (2017).

doi:10.1080/1061186X.2017.1339196

85. Acquati, F. *et al.* Microenvironmental control of malignancy exerted by RNASET2, a widely conserved extracellular RNase. *Proc. Natl. Acad. Sci. U. S. A.* **108,** 1104–9 (2011).

86. Acquati, F. *et al.* Loss of function of Ribonuclease T2, an ancient and phylogenetically conserved RNase, plays a crucial role in ovarian tumorigenesis. *Proc. Natl. Acad. Sci.* 1222079110- (2013). doi:10.1073/pnas.1222079110

87. Gabrielsen, I. S. M. *et al.* Genetic risk variants for autoimmune diseases that influence gene expression in thymus. *Hum. Mol. Genet.* ddw152 (2016). doi:10.1093/hmg/ddw152

88. Chu, X. *et al.* A genome-wide association study identifies two new risk loci for Graves' disease. *Nat. Genet.* **43,** 897–901 (2011).

89. Caputa, G. *et al.* RNASET2 is required for ROS propagation during oxidative stress-mediated cell death. *Cell Death Differ.* **23,** 347–57 (2016).

90. Wang, Q. *et al.* Stress-induced RNASET2 overexpression mediates melanocyte apoptosis via the TRAF2 pathway in vitro. *Cell Death Dis.* **5,** e1022 (2014).

91. Moret-Tatay, I. *et al.* Possible Biomarkers in Blood for Crohn's Disease: Oxidative Stress and MicroRNAs—Current Evidences and Further Aspects to Unravel. *Oxid. Med. Cell. Longev.* **2016,** 1–9 (2016).
